# Technical Education

**Published:** 1897-06-26

**Authors:** 


### The Hospital

A Journal of

The Medical Sciences and Hospital Administration.

Vol. XXII.—No. 561.] [June 26, 1897.

### Technical Education.

The Congress on Technical Education, which was held last week at the house of the Society of Arts, under the joint auspices of the Society and of some of the great City Companies, and with the Duke of Devonshire as its President, afforded to many doctrinaires an excellent opportunity of bringing forward their respective plans for the regeneration of the world. We have for a long time past been told that the one great requirement of the English working man is improved technical education, and that the extent to which this is given in Germany and other countries is the chief cause of the power of those countries to undersell us in markets which are open to all competitors. Now education, whether technical or general, can only be profitably conducted by the light of experience or by the light of physiology; and in this, as in so many other matters, it will often be found that experience has hit the nail on the head, long before physiology has come in to explain in what way the desired effect follows from giving a particular direction to the blow. There can be little doubt that what is commonly known as technical education has been comparatively neglected in England, nor can there be any doubt at all, in spite of the deficiency, that English work, in many departments of industry, has been and is the very best in the world. To take only a few examples, English watches, English porcelain, English table glass, are not only unequalled in any other country, but they are unapproached; and the sole reason why they are not in universal demand is that the education required is not the education of the producer to do better, but the education of the consumer to know good things from bad ones when he sees them.

Sir Joshua Fitch, whose long experience in educational matters entitles his opinions to very respectful consideration, presented to the Congress a paper which was only read in abstract, but which will no doubt be found, when published *in extenso*, to be eminently suggestive and instructive. He practically asked the Congress whether technical education in the strict sense—education to conduct a process or to follow a handicraft—could be profitably conducted apart from education of the faculties generally. We share his doubt; but, nevertheless, if we approach the question from a physiological standpoint, we shall see much reason to believe that muscular education of the extremities is attended by an increased development of the brain as a whole. Dr. Poore has lately raised the very interesting question whether the more frequent ambidexterity of women than of men, largely the consequence of the education of the left hand in piano-playing, may not tend to raise the motor centres of the right half of the brain to the functional level of those of the left half, and may not thus explain the greater fluency of speech which he declares to be characteristic of the female sex. We gravely doubt, however, whether the alleged deficiency in technical education of the English artisan be really the explanation of any of the trade difficulties with which he, or more frequently his employer, is called upon to contend. Those difficulties are mainly due to deficient education in other ways, to deficient education in the effects of conduct, and to deficient education of the judgment. The snare of English industry is the tendency of the artisan to "strike" when work is good, and to howl when it is bad. Both tendencies alike depend upon ignorance of the conditions which govern the prosperity of his trade, and upon readiness to be led by the nose by every blatant agitator who prefers talking to labour. The eminent physician to Rugby School, Dr. Dukes, has lately written that the education of the children of the upper and middle classes of this country is the only occupation for which those who pursue it are left utterly untrained; and we think he might have added that of the lower classes also. Of course, we know that there are "training colleges" for the teachers, but what does the training amount to. It undoubtedly assists those who receive it to prepare school children for passing in the respective "standards;" but do these standards represent any education which, in the language of Paley, is really a "preparation for the sequel of the lives" of those who learn. The unphysiological so-called "educationist" generally labours under the delusion that the introduction of compressed facts into a child's memory will mechanically expand his intellect; and forgets, if he ever knew, that any high intellectual development must, as a rule, be the cumulative result of the education of many generations. If we could push this person and his congeners into the background, could strike out a large proportion of the School Board subjects from the programme, and could teach the principles of conduct to the boys, and domestic economy to the girls, we are much disposed to believe that the technical education of English handicraftsmen might with great safety be left to take care of itself, in reliance upon the hereditary skill which is the outcome of hereditary industry. But to effect this change the doctor must intervene, in order to make it felt that the development of bodily functions must be based upon a careful study of the physiology of the organs which are subservient to them.

